# Risk factor contributions to socioeconomic inequality in cardiovascular risk in the Philippines: a cross-sectional study of nationally representative survey data

**DOI:** 10.1186/s12889-023-15517-x

**Published:** 2023-04-12

**Authors:** Callum Brindley, Tom Van Ourti, Joseph Capuno, Aleli Kraft, Jenny Kudymowa, Owen O’Donnell

**Affiliations:** 1 Erasmus School of Health Policy and Management, Erasmus University, 1738, Rotterdam, 3000 DR The Netherlands; 2 Erasmus Centre for Health Economics, Erasmus University Rotterdam, Rotterdam, the Netherlands; 3 Erasmus School of Economics, Erasmus University Rotterdam, Rotterdam, the Netherlands; 4 Tinbergen Institute, Amsterdam, the Netherlands; 5 School of Economics, University of the Philippines Diliman, Diliman, The Philippines; 6 Rethink Priorities, Frankfurt, Germany; 7 Faculty of Economics and Business, University of Lausanne, Lausanne, Switzerland

**Keywords:** Cardiovascular disease, Risk factors, Blood pressure, Blood glucose, Cholesterol, Smoking, Socioeconomic, Inequality, Decomposition, Philippines

## Abstract

**Background:**

Primary prevention of cardiovascular diseases (CVD) increasingly relies on monitoring global CVD risk scores. Lack of evidence on socioeconomic inequality in these scores and the contributions that specific risk factors make to this inequality impedes effective targeting of CVD prevention. We aimed to address this evidence gap by measuring and decomposing socioeconomic inequality in CVD risk in the Philippines.

**Methods:**

We used data on 8462 individuals aged 40–74 years from the Philippines National Nutrition Survey and the laboratory-based Globorisk equation to predict 10-year risk of a CVD event from sex, age, systolic blood pressure, total cholesterol, high blood glucose, and smoking. We used a household wealth index to proxy socioeconomic status and measured socioeconomic inequality with a concentration index that we decomposed into contributions of the risk factors used to predict CVD risk. We measured socioeconomic inequalities in these risk factors and decomposed them into contributions of more distal risk factors: body mass index, fat share of energy intake, low physical activity, and drinking alcohol. We stratified by sex.

**Results:**

Wealthier individuals, particularly males, had greater exposure to all risk factors, with the exception of smoking, and had higher CVD risks. Total cholesterol and high blood glucose accounted for 58% and 34%, respectively, of the socioeconomic inequality in CVD risk among males. For females, the respective estimates were 63% and 69%. Systolic blood pressure accounted for 26% of the higher CVD risk of wealthier males but did not contribute to inequality among females. If smoking prevalence had not been higher among poorer individuals, then the inequality in CVD risk would have been 35% higher for males and 75% higher for females. Among distal risk factors, body mass index and fat intake contributed most to inequalities in total cholesterol, high blood sugar, and, for males, systolic blood pressure.

**Conclusions:**

Wealthier Filipinos have higher predicted CVD risks and greater exposure to all risk factors, except smoking. There is need for a nuanced approach to CVD prevention that targets anti-smoking programmes on the poorer population while targeting diet and exercise interventions on the wealthier.

**Supplementary Information:**

The online version contains supplementary material available at 10.1186/s12889-023-15517-x.

## Key message

• Evidence on risk factor contributions to socioeconomic inequalities in cardiovascular disease (CVD) risk is needed to target prevention programmes and meet the SDG target of a one third reduction in premature non-communicable disease mortality by 2030.
• In the Philippines, wealthier individuals have higher predicted CVD risks and greater exposure to all risk factors, except smoking.
• Total Cholesterol (TC) and High Blood Glucose (HBG) contribute most to the higher CVD risks of wealthier Filipinos.
• Wealth inequalities in TC and HBG, and (for males) in systolic blood pressure, are mostly explained by differences in body mass index and fat share of energy intake, and not by differences in physical exercise.
• To maximise impact, CVD prevention in the Philippines should target diet programmes on wealthier groups and smoking programmes on poorer groups.

**Supplementary Information:**

The online version contains supplementary material available at 10.1186/s12889-023-15517-x.

## Introduction

Low-cost primary prevention interventions can reduce the large and accumulating burden of cardiovascular disease (CVD) in low- and middle-income countries (LMICs). [1–3] Realisation of this potential requires targeting high risks among people who are asymptomatic, have not previously experienced a CVD event, and yet account for around three fifths of all CVD deaths [4]. There has been a shift in primary prevention away from management of separate CVD risk factors, such as hypertension, dyslipidemia, high blood glucose, and smoking, to the assessment and control of CVD risk predicted from these factors [3, 5–9]. And yet there is no evidence from LMICs, or even from high-income countries, [10] on socioeconomic inequality in predicted CVD risk broken down into the contributions of separate risk factors. This impedes effective implementation of a risk-based primary prevention strategy because information is lacking to plan the targeted distribution of treatments that meet the needs of high-risk groups.

The relatively few LMIC studies that have examined associations between CVD risk and markers of socioeconomic status (SES) have delivered mixed evidence on the direction of the gradient [11–15]. More plentiful LMIC evidence on socioeconomic inequalities in separate CVD risk factors tends to show that higher SES groups are more exposed to most risk factors, [11, 13, 16–26] although this is not a universal finding [15, 27–29]. The opposite socioeconomic gradient is usually found for smoking tobacco [15, 16, 23, 24, 26, 30] and insufficient consumption of fruit and vegetables [16, 23, 30].

In the Philippines, the burden of CVD – measured by age-standardised disability-adjusted life years – has increased relatively from 41% below the average for Southeast Asia in 1990 to 10% above that average in 2019. [31] If this trend continues, the Philippines will not reach the Sustainable Development Goal (SDG) target of a one-third reduction in premature mortality from non-communicable diseases (NCDs) until 2050. [32] Modelling suggests that the Philippines, along with 19 other high CVD burden countries, could reach the SDG target by 2030, as scheduled, through investment, at relatively low cost, in the World Health Organization’s package of essential NCD interventions (PEN). [33, 34] Evidence on contributions to the socioeconomic pattern of CVD risk could improve targeting and so help close gaps in the implementation of the PEN, [34, 35] which includes risk-based primary prevention of CVD, by better matching the distribution of programme interventions, particularly medicines, to the distribution of need.

This study aimed to measure socioeconomic inequality in CVD risks faced by Filipino men and women aged 40–74 years and to explain the inequality by estimating contributions of the proximate risk factors used to predict CVD risk: age, raised blood pressure, high total cholesterol, high blood glucose, and smoking. As far as we know, this is the first study to make such a decomposition of socioeconomic inequality in CVD risk. We also aimed to identify contributions of more distal risk factors to socioeconomic inequalities in proximate risk factors.

## Methods

### Sample design and selection

We used data from the nationally representative National Nutrition Survey (NNS) conducted from June 2013 to April 2014 in all regions and provinces of the Philippines [36]. This stratified, multi-stage sample survey had a response rate of 91.3% at the household level and 91.5% at the individual level (Supplementary Text S1 for sample design) [36]. Partially overlapping sub-samples of participants were randomly selected for blood tests, anthropometric and blood pressure measurements, and completion of a health interview. Response rates for these components ranged from 83–96% [36]. Th 2013 NNS was the last one with complete data on fasting blood sugar and lipid profile.

We restricted the analysis sample to participants aged 40 to 74 years who were included in the sub-samples randomly selected for measurement of risk factors used to predict CVD risk. The lower age limit was necessitated by the CVD risk prediction equation we used. The upper age limit was imposed to avoid inclusion of older people with many comorbidities. We also excluded participants who reported either having been diagnosed with CVD or having experienced symptoms of myocardial infarction or stroke (sudden slurring of speech, or weakness or numbness in part of the body that lasted more than 24 h). [37] For secondary analysis that sought to explain socioeconomic inequality in proximate physiological risk factors, we used a subset of the analysis sample that was restricted to participants who were randomly selected to provide data on body mass index (BMI), physical activity, fat intake, and alcohol consumption, in addition to the previously mentioned risk factors.

### Measurements

Household and individual sociodemographic characteristics were reported in face-to-face interviews. Nurses used a non-mercurial sphygmomanometer and stethoscope to measure blood pressure following standard procedure (Supplementary Text S2). After a participant had fasted overnight for 10–12 h, a registered medical technologist used venipuncture to draw a blood sample that was analysed to determine total cholesterol (TC) (mmol/L) and fasting blood sugar (FBS) (mg/dL). We excluded participants with biologically implausible values of systolic blood pressure (SBP) (< 70 or > 270 mmHg), TC (< 1.75 or > 20 mmol/L), or FBS (< 30 or > 600 mg/dL) [37]. Health professionals measured weight and height following standard procedures (Text S2). We calculated BMI from these measurements.

Participants in random sub-samples reported health behaviours. We classified those who reported currently smoking tobacco as *smokers*. We defined those who reported having at least one alcoholic beverage in the past 30 days as *alcohol drinkers*. Using responses to the WHO Global Physical Activity Questionnaire, we defined *low physical activity* as metabolic equivalent activity less than 600 min per week [38]. Using the 24-hour Food Recall method, we calculated *fat intake* as a percentage share of total dietary energy for each participant (Text S2).

We measured SES by a household *wealth index* that was the first principal component of a factor analysis of ownership of durable assets, transport mode, housing type and materials, water source, sanitation, and electricity supply (Text S2) [39]. Compared with educational attainment, the advantages of using a wealth index to proxy SES are that it (a) captures more information, (b) focuses more on living standards, (c) differentiates more between individuals, and (d) is more comparable between differently aged individuals.

### Outcome

We used the *Globorisk* laboratory-based Eq. 3[7] to predict the probability of experiencing, within 10 years, a fatal or non-fatal CVD event, defined as (a) death from ischaemic heart disease or sudden cardiac death, (b) death from stroke, or (c) non-fatal myocardial infarction or stroke. We used the Philippines-calibrated equation to predict this CVD risk of each participant using data on their age, sex, SBP, TC, FBS, and smoking status (Supplementary Text S3). The equation, which is valid at ages of 40 years and older, used diabetes status, which we proxied by FBS ≥ 126 mg/dL and refer to as high blood glucose (HBG) [40]. We also created a binary indicator of *high CVD risk*, defined as CVD risk ≥ 20% [41–43].

### Statistical analysis

We stratified all analyses by sex. In supplementary analyses, we also stratified by urban/rural. For some analyses, we used the wealth index to categorize participants into quintile groups. Across these groups, we compared means of CVD risk and the proximate risk factors. We used a concentration index – a scaled covariance – to quantify inequality in CVD risk over the full wealth index distribution (Supplementary Text S4). [44, 45] A positive (negative) value of the concentration index indicates that wealthier (poorer) participants had higher CVD risk. We used an index that measures absolute inequality and is related to the slope index of inequality [46].

We used the Shapley value decomposition [47, 48] to apportion the concentration index measure of inequality in CVD risk into contributions of the proximate risk factors (Supplementary Text S5). We identified the contribution of a risk factor by estimating the change in the concentration index generated by the elimination of inequality in that risk factor. To deal with path dependence arising from nonlinearity of the risk prediction equation, we calculated the contribution of a risk factor for each possible sequence of eliminating inequality in all risk factors. We then averaged the contributions of that risk factor over all possible sequences. We eliminated inequality in SBP, TC, HBG, and smoking by setting each of these risk factors to its age- and sex-specific mean [47]. The contributions of these risk factors were effectively age-standardised in a way took account of the nonlinearity of the risk equation and interactions between age and the risk factors. To get the contribution of age, we subtracted the sum of the contributions of the other risk factors from the concentration index for CVD risk. The index minus the age contribution gives Age-standardized inequality.

A positive (negative) contribution of a risk factor indicated that it caused wealthier (poorer) individuals to have higher predicted CVD risk. The direction and magnitude of each contribution depended on socioeconomic inequality in the respective risk factor, the effect of the risk factor on predicted CVD risk, and the extent to which that effect differed along the wealth index distribution due to interactions with other wealth-varying risk factors that were captured by the nonlinear risk prediction equation. We used the concentration index to measure inequality in each risk factor. We used the Globorisk equation to obtain the average marginal effect (AME) on predicted risk of an age- and sex-specific one standard deviation increase in each risk factor. We also averaged the marginal effects within each wealth index quintile group to examine differences arising from multiplicative effects of risk factors combined with differences in their levels and composition by wealth.

We decomposed inequality in predictions of the three physiological risk factors – SBP, TC, and HBG – into contributions of more distal risk factors – BMI, low physical activity, fat intake, and drinking alcohol. We modelled each of SBP, TC, and HBG as a function of the distal risk factors and age. For SBP and TC, we used a generalised linear model (GLM) with a Poisson distribution and a log link function. For HBG, we used logit. We used the estimates to predict SBP, TC, and the probability of HBG. We used the concentration index [44] to measure socioeconomic inequality in each predicted physiological risk factor and decomposed the respective index into the contributions of the four distal risk factors and age using the Shapley value decomposition [47].

We applied sample weights in all analyses. We adjusted the weights for selection into the complete cases sample (Supplementary Text S6). [49] We adjusted confidence intervals to take account of sample stratification and clustering. We used a bootstrap with 1000 repetitions to obtain confidence intervals for the decomposition estimates.

## Results

### Participant characteristics

From a sample of 51 771 individuals of aged 40–74 years in the core 2013 NNS, we obtained a primary analysis sample of 8462 individuals who were randomly selected for survey modules used to predict CVD risk and had complete response on requisite items (Supplementary Figure S1). This sample was used to measure and decompose inequality in CVD risk. A secondary analysis sub-sample of 2900 individuals who were randomly selected for survey modules providing data on the distal risk factors was used to explain inequality in SBP, TC, and HBG (Figure S1). Within the selected age interval and after the application of weights, characteristics of both analysis samples were very similar to the core NNS survey sample (Supplementary Table S1).

**Table 1:** Participant characteristics

		Primary analysis sample				Secondary analysis sample			
		Female, n = 4516		Male, n = 3946		Female, n = 1801		Male, n = 1099	
CVD risk, %	mean (SD)	12.4	(11.5)	17.1	(11.1)	12.3	(11.0)	17.4	(11.7)
High CVD risk ≥ 20%	n (%)	871	(18.3)	1,276	(31.8)	375	(18.5)	392	(31.5)
Age, years	mean (SD)	52.7	(9.2)	52.1	(8.8)	52.7	(9.2)	52.1	(8.8)
Systolic blood pressure (SBP), mmHg	mean (SD)	124.4	(20.8)	126.4	(19.8)	124.9	(21.1)	126.8	(21.1)
High SBP ≥ 140 mmHg	n (%)	1,364	(29.3)	1,312	(33.3)	555	(30.0)	383	(33.5)
Total cholesterol (TC), mmol/L	mean (SD)	5.7	(1.2)	5.2	(1.2)	5.7	(1.2)	5.3	(1.2)
High TC > 6.2 mmol/L	n (%)	1,366	(29.8)	688	(19.1)	582	(31.3)	203	(19.3)
Fasting blood sugar (FBS), mg/dL	mean (SD)	98.3	(39.0)	99.8	(39.5)	97.3	(37.8)	99.3	(37.5)
High blood glucose ≥ 126 mg/dL	n (%)	371	(8.3)	314	(8.9)	137	(7.5)	102	(9.3)
Smoker, %	n (%)	414	(8.5)	1,797	(45.1)	145	(7.5)	468	(43.5)
Wealth index quintile group	n (%)								
Poorest		1,073	(20.0)	967	(20.0)	420	(20.0)	251	(20.1)
Poor		993	(20.0)	876	(20.0)	382	(20.0)	223	(20.0)
Middle		899	(20.0)	801	(20.0)	359	(20.0)	226	(20.0)
Rich		811	(20.0)	701	(20.0)	339	(19.9)	210	(19.9)
Richest		740	(19.9)	601	(20.0)	301	(20.0)	189	(20.0)
Urban location	n (%)	1,957	(52.4)	1,571	(50.7)	826	(52.2)	509	(50.8)
Body mass index (BMI), kg/m2	mean (SD)					24.2	(4.6)	23.2	(3.8)
Overweight, BMI ≥ 25	n (%)					682	(41.1)	314	(29.5)
Low physical activity	n (%)					1,018	(55.6)	569	(52.4)
Fat share energy intake, %	mean (SD)					14.9	(8.6)	13.8	(8.4)
Drinks alcohol	n (%)					207	(11.1)	535	(50.1)

*Note.* The primary analysis sample was used to measure and decompose inequality in CVD risk. The secondary analysis sample was used to measure and decompose inequalities in SBP, TC, and HBG into contributions of BMI, low physical activity, fat intake, and drinking alcohol. See Supplementary Figure S1 for selection of the respective samples. Frequencies are unweighted. Means and percentages are weighted

Table 1 shows characteristics of both analysis samples stratified by sex. The average age was around 52 years. The samples were approximately evenly split between urban and rural locations. Around 30% of participants had high SBP (≥ 140 mmHg). High TC (> 6.2 mmol/L) was measured in around 30% of female participants and 19% of male participants. Approximately 9% of participants had HBG (≥ 126 mg/dL).

### Disparities in CVD risk and proximate risk factors

**Fig. 1:**
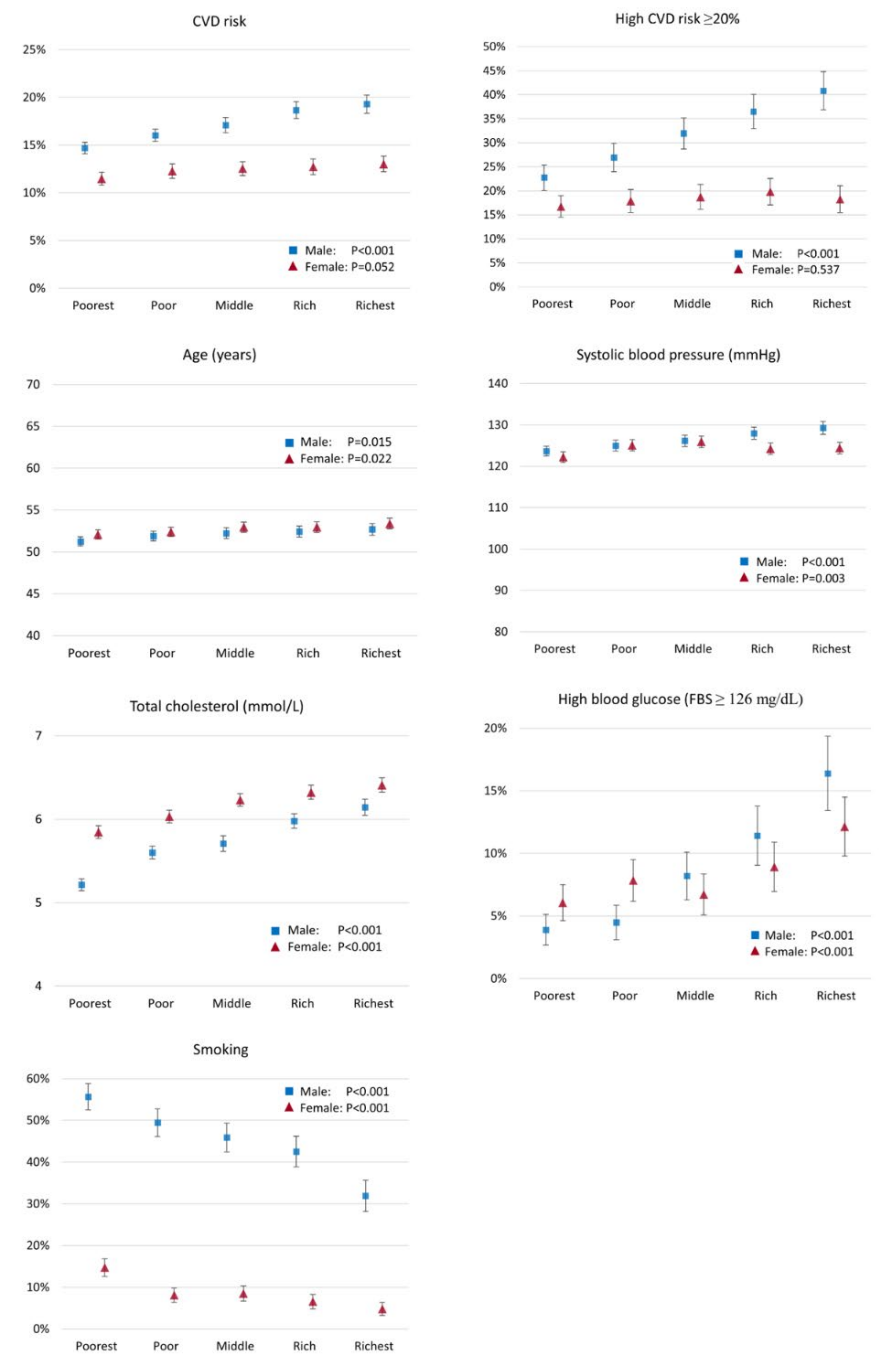
Mean CVD risk and risk factors by wealth index quintile group and sex. Individuals aged 40-74 years. Females: n=4516. Males: n=3946. Whiskers show 95% confidence intervals. P values from tests of equal means across groups

Figure 1 shows means for risk of a fatal or non-fatal CVD event within 10 years and the risk factors used to predict this risk by wealth index quintile group. For males, mean CVD risk was 14.7% (95% CI: 14.1%, 15.3%) among the poorest fifth, compared with 19.3% (95% CI: 18.3%, 20.3%) among the richest fifth. The socioeconomic gradient in the prevalence of high CVD risk (≥ 20%) was even steeper. For females, the socioeconomic gradient in each outcome was much less pronounced than the respective gradient for males. The respective socioeconomic gradient in the risk of a fatal CVD event was smaller absolutely but not relatively (Supplementary Figure S2). The means of most of the CVD risk factors were higher in wealthier groups, particularly for males. Smoking showed the reverse gradient.

**Fig. 2:**
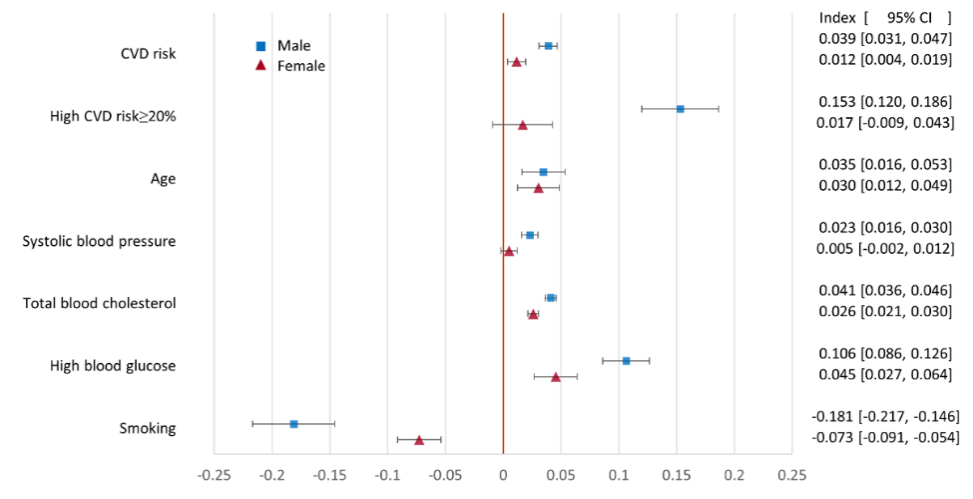
Concentration index measure of socioeconomic inequality in CVD risk and risk factors by sex. Females: n = 4516. Males: n = 3946. Whiskers show 95% confidence intervals

All point estimates of the concentration indices for CVD risk and the proximate risk factors, except for smoking, are positive (Fig. 2), indicating that wealthier individuals had higher risks. With the exceptions of high CVD risk and SBP for females, the 95% confidence intervals do not include zero. For both sexes, the concentration index for smoking is negative, indicating that prevalence was higher among poorer individuals. For all risks and risk factors, except age, the point estimates and 95% confidence intervals indicate greater socioeconomic inequalities among males compared to females.

### Decomposition of inequality in CVD risk

**Fig. 3:**
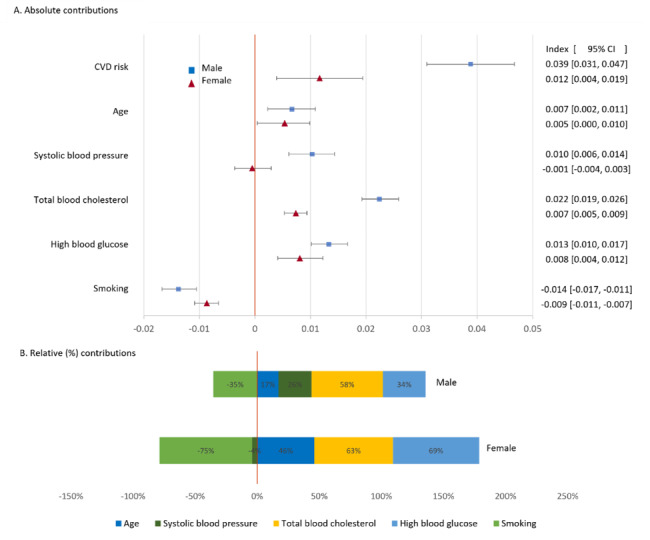
Decomposition of concentration index measure of socioeconomic inequality in CVD risk into contributions of risk factors. Top panel shows absolute contributions. Bottom panel shows these contributions as percentages of the concentration index. Females: n = 4516. Males: n = 3946. Whiskers show 95% confidence intervals obtain from a bootstrap with 1000 replications

Figure 3 shows the concentration index of CVD risk followed by its decomposition into the contributions of the risk factors. The top panel shows absolute contributions. For each sex, these sum to the respective concentration index for CVD risk. The bottom panel shows the relative contributions that sum to 100%.

For both sexes, age contributed to the higher CVD risk of wealthier individuals. This is because risk increased with age and wealthier people tended to be older (Figs. 1 and 2). Age accounted for 17% and 46% of the socioeconomic inequality in CVD risk for males and females, respectively. For males, the distribution of SBP accounted for about a quarter of the inequality in CVD risk that is to the disadvantage of wealthier individuals. SBP did not contribute to the higher CVD risk of wealthier women. TC and HBG accounted for 58% and 34%, respectively, of the socioeconomic inequality in CVD risk for males, and 63% and 69%, respectively, for females. For both sexes, smoking had a large offsetting effect – 35% for males and 75% for females – that pushed inequality in the direction of higher risk for poorer individuals.

**Fig. 4:**
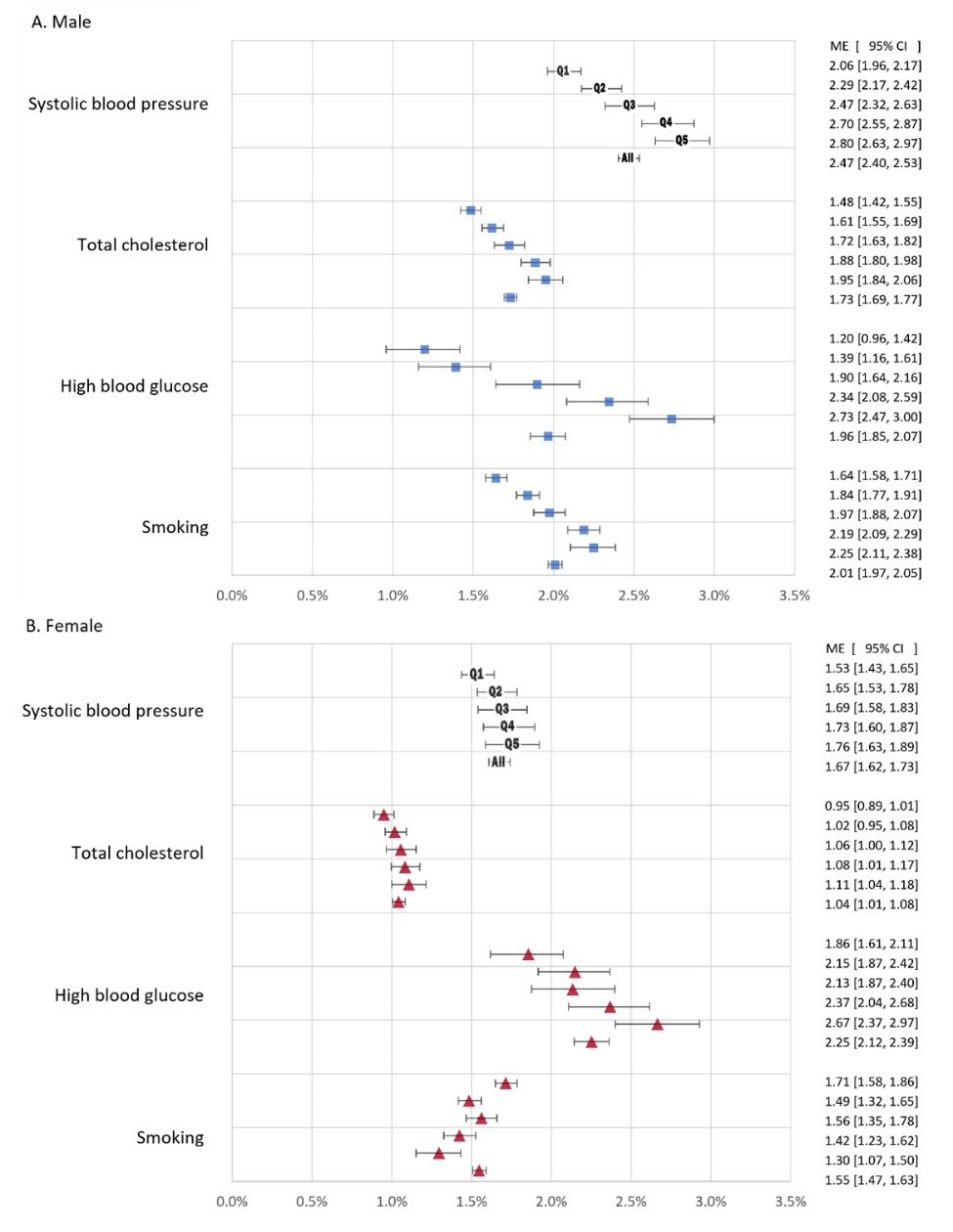
Average marginal effect of an age- and sex-specific standard deviation increase in each risk factor on CVD risk averaged over the sample and within each wealth index quintile group by sex. Females: n = 4516. Males: n = 3946. Whiskers show 95% confidence intervals obtain from a bootstrap with 1000 replications. Q1 = poorest 20%,…, Q5 = richest 20%. All = Average over all individuals

Figure 4 shows risk equation estimates of the AME of each risk factor on predicted CVD risk for the population and each wealth group. For males, a standard deviation increase in each of HBG and smoking raised the predicted CVD risk by about 2% points (pp). The AME of a standard deviation increase in SBP was larger, while that of TC was smaller. For females, the largest AME was from HBG, followed by SBP, smoking, and TC. The AME of each risk factor, except for smoking among females, was larger in wealthier groups, implying a more detrimental composition of risk factors in these groups.

To facilitate comparison of effects across risk factors, we show the AME of a standard deviation increase in each. When we scaled the AME by the range of each risk factor, which is more relevant to the contribution each makes to the concentration index measure of CVD risk inequality, then TC had the largest and most heterogeneous effect (Supplementary Table S2). That is why TC made the largest contribution to CVD risk inequality for males.

Mean CVD risk was higher in urban than in rural locations, particularly for males (Supplementary Table 3). For both sexes, the excess CVD risk of wealthier compared with poorer individuals was greater in rural areas (Supplementary Figure S3). This was due to larger contributions of SBP and, particularly, TC to the socioeconomic inequality in rural areas (Figure S3).

### Decomposition of inequality in physiological CVD risk factors

**Fig. 5:**
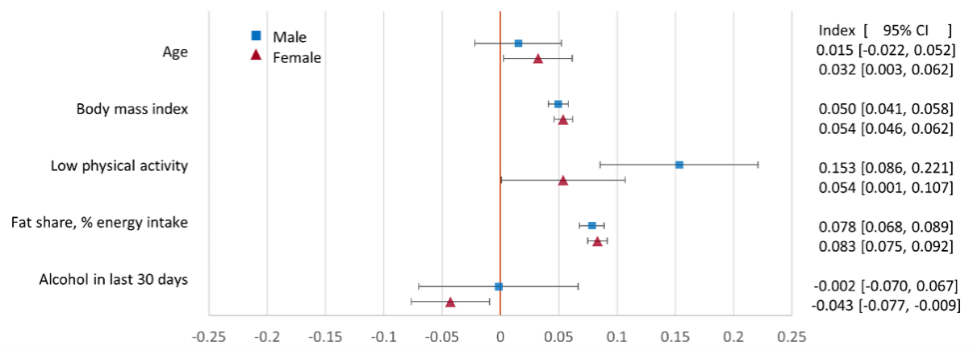
Concentration index measure of socioeconomic inequality in distal risk factors and age by sex. Females: n = 1801. Males: n = 1099. Whiskers show 95% confidence intervals

The positive concentration indices for BMI, low physical activity, and fat share of energy intake shown in Fig. 5 indicate that wealthier individuals were more exposed to each of these distal risk factors. Alcohol consumption displayed no socioeconomic inequality among males and was more prevalent among poorer females.

**Fig. 6:**
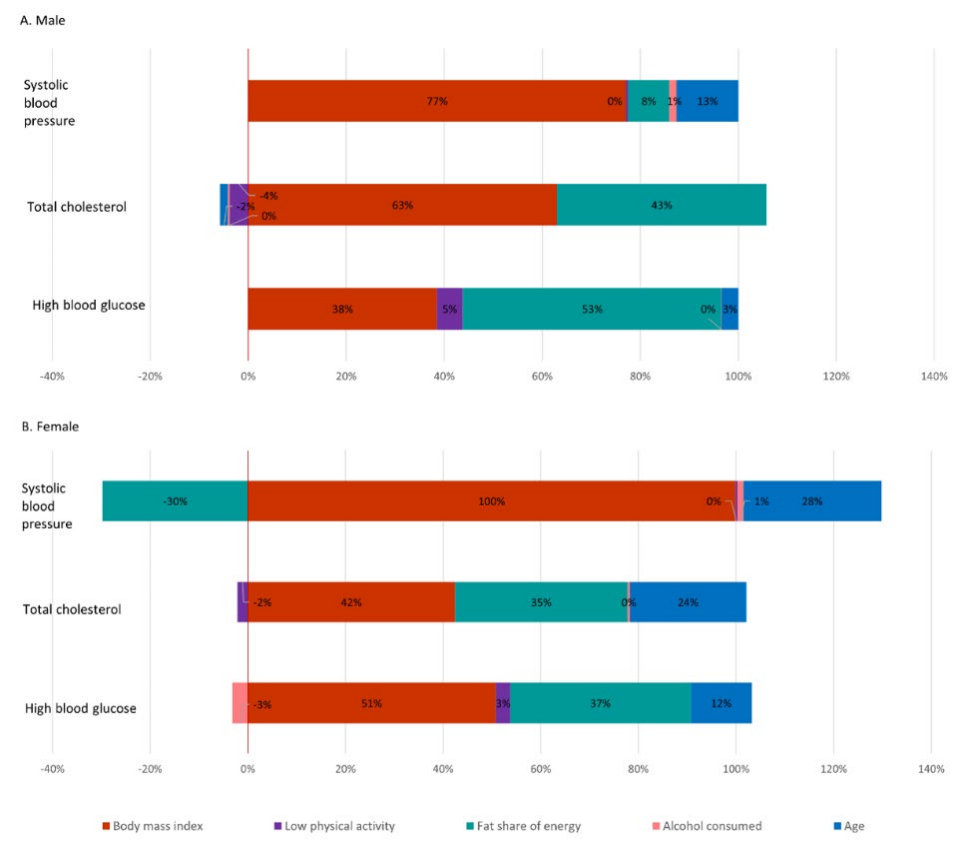
Decomposition of concentration index measure of socioeconomic inequality in predicted SBP, TC, and HBG into relative (%) contributions of distal risk factors. Females: n = 1801. Males: n = 1099

Figure 6 shows relative contributions of the distal risk factors to socioeconomic inequality in predictions of each of SBP, TC, and HBG (Supplementary Figure S4 for absolute contributions). BMI made the largest contribution to inequality in each of these three physiological risk factors, with HBG being a slight exception for males. Fat share of energy intake also contributed substantially to higher TC levels and to higher HBG prevalence experienced by wealthier individuals. Fat intake contributed to higher SBP among poorer females, which was due to an unexpected negative estimated association between SBP and fat intake (Supplementary Table 4). Low physical activity and alcohol made relatively small contributions to the inequalities that never exceeded 5%. Age differences contributed substantially to the higher levels of SBP, TC, and HBG of wealthier women. For men, the age contribution was more modest, and slightly offsetting in the case of TC.

## Discussion

We used nationally representative data on 8462 Filipinos aged 40–74 years to measure sex-specific socioeconomic inequality in the 10-year risk of a cardiovascular event predicted with the laboratory-based Globorisk equation. We decomposed this inequality into contributions of risk factors: age, systolic blood pressure (SBP), total cholesterol (TC), high blood glucose (HBG), and smoking. We found that wealthier Filipinos had higher CVD risks, particularly among males. This could signal potential for a further rise in the CVD burden, which is already relatively high in the Philippines, [33, 50] as the country develops economically. Development may also lead to weakening of the gradient, or even its reversal [15, 23, 24, 30, 51].

All proximate risk factors, with the exceptions of smoking for both sexes and SBP for females, contributed to wealthier individuals facing higher CVD risks. This was due to wealthier individuals being more exposed to each risk factor, with the noted exceptions, and because the predicted CVD risks of these individuals responded more to a marginal increase in each risk factor, except for the effect of smoking on females. The latter heterogeneity was because greater exposure to one risk factor increased the marginal effects of others. For both sexes, TC and HBG contributed most to wealthier individuals having higher CVD risks. Eliminating inequality in these risk factors would leave little or no socioeconomic difference in CVD risk among males and, due to the countervailing effect of smoking, would leave poorer females facing a higher risk of CVD. If smoking had not been more prevalent among poorer individuals, then inequality in CVD risk to the disadvantage of the wealthier would have been more than a third greater among males and three quarters greater among females.

These findings point to the need for nuanced prevention policies that combine use of a global risk score to identify high risks with targeting of specific interventions on populations depending on the factors contributing to high risk. To maximise the impact of smoking prevention programmes on CVD risk in the Philippines, they should be targeted at poorer groups. Interventions to reduce levels of cholesterol and blood sugar can have their greatest impact by targeting wealthier individuals.

For both sexes, we found that inequalities in predicted TC and HBG were mostly explained by inequalities in BMI and fat share of energy intake. BMI was also the largest contributor to the socioeconomic gradient in predicted SBP. The lower physical activity of wealthier individuals contributed much less to their greater exposure to proximate, physiological risk factors for CVD. Interventions that focus on diet, as opposed to exercise, may therefore be best placed to target excess risks of higher SES groups in the Philippines, although it is not possible to infer causal effects of such interventions from the statistical associations we estimated.

Our finding of higher CVD risk among wealthier Filipinos is consistent with evidence from India [13] and Tunisia [12]. But an analysis of 45 LMICs did not find a consistent sign for the (partial) association between CVD risk and wealth [15]. We found that not only was the average CVD risk higher for males than for females in the Philippines, as has been found in many other LMICs, [12, 13, 15, 41, 52–54] but there was also a steeper socioeconomic gradient in the CVD risks of males. This differs from evidence from India [13]. Consistent with what was found in India, [13] we established that CVD risks were lower but displayed greater socioeconomic inequality in rural parts of the Philippines.

Our finding that wealthier Filipinos had higher (systolic) blood pressure is consistent with evidence on the socioeconomic gradient in the prevalence of hypertension obtained from a systematic review of LMIC studies [17], multi-LMIC studies, [19, 21] and individual LMIC studies [13, 16, 18, 20]. There is, however, evidence of a gradient in the opposite direction in some LMICs, [27–29] and a multi-country study delivered mixed evidence on the direction of this gradient [15]. In common with our finding that wealthier Filipinos had higher TC levels, all but one [27] of the relatively few LMIC studies that examined socioeconomic inequality in high cholesterol found higher prevalence among wealthier individuals [11, 16, 19, 22]. Similarly, our finding that HBG was more prevalent among wealthier Filipinos is consistent with most of the LMIC evidence, [11, 13, 16, 18–20, 22, 25] with the same exception [27]. Higher prevalence of smoking among poorer Filipinos is consistent with the predominant finding from LMICs [15, 16, 23, 26, 30]. Our findings that wealthier Filipinos had higher BMI and lower physical activity are consistent with evidence from multi-country studies [15, 26, 23, 24, 30].

The main strength of this study is that it went beyond measurement of socioeconomic inequality in CVD risk and inequalities in separate risk factors to identify contributions the latter made to the former. Each contribution did not depend only on inequality in the respective risk factor but also on its effect on predicted CVD risk and how that differed by wealth due to interactions with other wealth-varying risk factors. Hence, our approach offers more insight than would be achieved by examining inequality in CVD risk alongside inequality in each risk factor. For males, the latter approach would have identified HBG as the risk factor that was most unequally distributed to the disadvantage of richer individuals and missed that TC makes the largest contribution to inequality in CVD risk. We identified contributions to variation in CVD risk that can be used to more effectively target policies that seek to shift primary prevention of CVD away from single risk factor management to assessment and control of global risk [3–9]. Nationally representative data on measured TC and FBS allowed us to predict CVD risk with a laboratory-based equation and avoid substantially underestimating risks of those indicated to have diabetes [55].

One limitation of the study is that the complete cases sample is substantially smaller than the core survey sample. However, the difference is mainly due to selection on age and random selection of participants for required survey modules. Selection due to item non-response was not high. A more important limitation is that the data were collected in 2013-14. More recent data were not available because the Covid-19 pandemic delayed the ongoing round of the National Nutrition Survey. The estimated contributions of risk factors depended on how the Globorisk equation weighted and combined them into a score. While this risk prediction equation that has not been validated for the population of the Philippines, it was purposefully designed to facilitate recalibration for use in multiple countries. The level of predicted CVD risk is known to depend on the risk equation used [13, 14, 56, 57]. This is inconsequential for our measurement and decomposition of CVD risk inequality because we used a measure of absolute inequality that is invariant to a level shift in the outcome. And there is LMIC evidence that the socioeconomic gradient in Globorisk-predicted CVD risk is similar to that obtained with other risk Eqs.  [13, 14] Globorisk predictions have been found to correlate strongly with those obtained from the Framingham [58] equation but less strongly with those from the WHO-CVD [41] equation [57]. It would have been preferable to establish robustness of our main findings to use of the WHO-CVD equation. We used a single measurement of FSB to indicate HBG, which is inconsistent with recommendations for clinical diagnosis of diabetes [59]. While this will have increased noise in the predictions of CVD risk, it avoided biases that would have arisen if reported diagnoses of diabetes had been used. A low estimated mean fat share of energy intake may have been due to bias in the 24-hour food recall method used. Our analysis was descriptive, not causal. To decompose inequality in predicted physiological risk factors, we estimated cross-sectional partial associations between physiological and distal risk factors. These estimates may be sensitive to the model specification. This would be limiting if the objective were to identify causal effects. It is not limiting for targeting of CVD prevention on the basis of associated risk factors.

We measured and decomposed socioeconomic inequality in predicted CVD risk and not in the incidence of CVD events. Disparities in the diagnosis, treatment, and control of CVD risk factors are generally found to be to the disadvantage of lower socioeconomic groups [21, 60–64]. Hence, while poorer individuals may be less exposed to these risk factors, with the exception of smoking, less access to preventive healthcare could possibly leave these individuals at greater risk of succumbing to CVD, as was found in the PURE cohort study [24]. Our findings give reason to target interventions that are intended to reduce risk factor exposure (other than smoking) on higher socioeconomic groups in the Philippines, but they do not support such targeting of primary prevention of CVD generally. Healthcare and pharmacological therapies aimed at controlling (as opposed to preventing) risk factors, such as hypertension and dyslipidaemia, may well have greater effect if targeted on lower socioeconomic groups.

This study undertook the first decomposition of socioeconomic inequality in CVD risk and revealed that higher risk among wealthier Filipinos was mainly due to socioeconomically skewed distributions of total cholesterol and high blood glucose. In turn, the inequalities in these proximate risk factors were mainly explained by wealthier individuals having higher BMI and fat intake.

## Electronic supplementary material

Below is the link to the electronic supplementary material.

Supplementary Material 1

## Data Availability

The authors were given remote server access to the data underlying this article by the Food and Nutrition Research Institute, Department of Science and Technology (FNRI-DOST), Republic of the Philippines. The authors do not have permission to share the data. Access to Public Use Files of the National Nutrition Survey can be obtained on application to FNRI-DOST: http://enutrition.fnri.dost.gov.ph/site/policy.php. The software code used to analyse the data underlying this article will be shared on reasonable request to the corresponding author.

## Declarations

### Patient and public involvment

Patients or the public were not involved in the design, or conduct, or reporting, or dissemination plans of our research.
