# Supplementary material for: Risk factor contributions to socioeconomic inequality in cardiovascular risk in the Philippines: a cross-sectional study of nationally representative survey data
Source: BMC Public Health. 2023 Apr 12;23:689. doi: 10.1186/s12889-023-15517-x (PMC10092926; doi:10.1186/s12889-023-15517-x)
Supplement: Supplementary file 1 — Supplementary Material 1 [file 12889_2023_15517_MOESM1_ESM.docx]

Risk factor contributions to socioeconomic disparity in cardiovascular risk in the Philippines: a cross-sectional study of nationally representative survey data

**Supplementary Material**

**Table of contents**

Supplementary Text S1. Sample design 2

Supplementary Text S2. Measurements 3

Supplementary TextS3. CVD risk equation 5

Supplementary TextS4. CVD risk concentration index 7

Supplementary TextS5. Decomposition of CVD risk concentration index 9

Supplementary Text S6. Adjustment to sample weights 11

Supplementary Figures 12

Supplementary Tables 16

**Supplementary Text S1. Sample design**

We used data from the nationally representative National Nutrition Survey (NNS) conducted from June 2013 to April 2014 in all 17 regions and 80 provinces of the Philippines.^1^ The survey was stratified by region and province. It had a three-stage sampling design. The primary sampling unit was a barangay (or contiguous barangays) with at least 500 households. A barangay is the smallest administrative unit in the country. From each randomly sampled barangay, an enumeration area (EA) consisting of 150-200 households was randomly sampled. Finally, 11-12 households were randomly sampled from each EA. A household was defined as a group of persons who may be related or not, who sleep in the same dwelling unit and have common arrangements for the preparation and consumption of food. The sampling frame of households was taken from the 2009 Labor Force Survey. All individuals in each sampled household were included in the survey.

**References**

1. Food and Nutrition Research Institute Department of Science and Technology (FNRI-DOST). *Philippine Nutrition Facts and Figures 2013: 8th National Nutrition Survey Overview*

**Supplementary Text S2. Measurements**

*Anthropometrics*. Standard procedures were followed to measure the weight and height of all participants.^1^ Weight was measured using a Detecto^TM^ platform beam balance weighing scale with 160-kilogram capacity. Two measurements were made. A third measurement was taken if the difference between the first two measurements was greater than 0.3 kg. We used the mean of the last two measures, recorded to the nearest 0.1 kg. Standing height was measured using a Seca^TM^ microtoise. Individuals with health issues that might affect physical measurements (e.g. polio, artificial limb) were not measured for height. Two measurements were obtained. A third measurement was taken if the difference between the first two measurements was greater than 0.5 cm. We used the mean of the last two measures, recorded to the nearest 0.1 cm.

*Blood pressure*. Following standard procedures, blood pressure (BP) was measured using an A&D UM-101^TM^ non-mercurial sphygmomanometer with digital display and a Kawe^TM^ dual stethoscope.^2^ The participant was seated with arms bared, supported, and at the heart level. He or she was instructed to rest for five minutes and not to smoke or ingest a caffeine-containing beverage within 30 minutes before measurement. The edge of the cuff was placed 2.5 centimeters above the elbow crease, with the bladder directly over the brachial artery. The bladder was inflated to 30 mmHg above the point of radial pulse extinction as determined by a preliminary palpatory determination (estimated systolic pressure). It was then deflated at a rate of 2 mmHg per second, with the stethoscope bell (funnel) placed directly over the brachial artery. Systolic pressure was recorded at the appearance of the 1st clear tapping sound (two consecutive tapping sounds) (Korotkoff Phase I). Diastolic blood pressure was recorded at the disappearance of these sounds (Korotkoff Phase V), unless these were still present at or near 0 mmHg (Absent Phase V), in which case, softening of the sounds or muffling was used as diastolic pressure (Korotkoff Phase IV). For every individual, two readings, taken at least one to two minutes apart, were recorded. If these readings differed by more than 4 mmHg, a third reading was taken. We used the mean systolic BP from the last two readings.

*Fat intake*. Following standard procedures, food consumption at the individual level was estimated using the 24-hour food recall method in a face-to-face interview on two non-consecutive days.^3^ Participants were asked to remember and report all foods and beverages consumed during the previous 24-hours. All food items consumed, as well as their description, including cooking method and brand names, were exactly recorded. The amounts consumed were quantified in terms of common household measurements such as cups, tablespoons, or by size and number of pieces. The weight of each food item was then computed from the recalled quantities using a database of food weights and measures that is maintained by the Philippine Food and Nutrition Research Institute (FNRI). Dietary energy in calories (kcal) and the respective contributions of carbohydrates, fats, and protein were obtained by converting the computed weight of each food item using FNRI’s Food Composition Tables.^4^ We computed *fat intake* as a percent share of total dietary energy and used the average of the two non-consecutive 24-hour recall measures.

*Wealth index*. We used the wealth index supplied by FNRI-DOST with the NNS data. This was obtained as the first principal component from analysis of the following items:

- Housing characteristics – dwelling unit type, house/lot tenure, roof type, wall type, floor type, number of bedrooms, fuel used, electricity supply;
- Transport used – bicycle, motorcycle, car, jeep, van, tractor, horse-drawn cart, boat;
- Ownership of appliances – computer/laptop/tablet, telephone, cellphone/smartphone, television, radio/cassette recorder, VCD/DVD Player, camera/video camera, refrigerator/freezer, stove/range/microwave oven, blender/food processor, electric generator, air-conditioner, washing machine, electric fan, sewing machine, piano/organ and wall clock;
- Presence of household help;
- Water and sanitation – source of drinking water, source of water for cooking and washing, type of toilet, and garbage disposal system.

**References**

1. Food and Nutrition Research Institute, Department of Science and Technology (FNRI-DOST) (2015a) *Philippine Nutrition Facts and Figures 2013: Anthropometric Survey*
2. Food and Nutrition Research Institute, Department of Science and Technology (FNRI-DOST) (2015b) *Philippine Nutrition Facts and Figures 2013: Clinical and Health Survey*
3. Food and Nutrition Research Institute, Department of Science and Technology (FNRI-DOST) (2015c) *Philippine Nutrition Facts and Figures 2013: Food Consumption Survey*
4. Food and Nutrition Research Institute, Department of Science and Technology (FNRI-DOST) (1997) *Philippine Food Composition Tables*

**Supplementary Text S3. CVD risk equation**

We used the *Globorisk* laboratory-based equation^1^ to predict the probability of a fatal or non-fatal CVD event within 10 years (CVD risk) for each participant. A CVD event was defined as death from ischaemic heart disease or stroke, a sudden cardiac death, or a non-fatal myocardial infarction or stroke. CVD risk was predicted from sex, age, systolic blood pressure (SBP), total cholesterol (TC), high blood glucose (HBG) (fasting blood sugar (FBS) ≥ 126 mg/dL), and smoking status. The equation was constructed from eight US prospective cohorts of individuals aged 40 years and older with no history of CVD. It was designed for application in countries other than the US after recalibration using country-specific CVD event incidence rates and age-sex-specific means of the risk factors that enter the equation.

The hazard rate in each year of the 10-year period for individual *i* with sex *s* (0=male, 1=female), age *t* in years, and with baseline risk factors $x_{1i}$ = SBP in mmHg, $x_{2i}$= TC in mmol/L, $x_{3i}$=1 if HBG and 0 otherwise, and $x_{4i}$=1 if reported smoking tobacco and 0 otherwise, was obtained from:

$\varphi_{i}\left( t \right)=\varphi_{s}\left( t \right)\exp\left[ \sum_{k=1}^{4} \beta_{k}(x_{ki}-\overline{x}_{kst})+ \sum_{k=1}^{4} \delta_{k}t(x_{ki}-\overline{x}_{kst})+\sum_{k=3}^{4} \gamma_{k}s_{i}(x_{ki}-\overline{x}_{kst}) \right]$, (1)

where $\varphi_{s}\left( t \right)$ is the average hazard rate of a CVD event for sex *s* at age *t* in the Philippines, $\beta_{k}$ is the log hazard ratio for the main effect of risk factor *k* that was estimated from a Cox proportional hazards model of the 10 US cohorts,^1^ $\delta_{k}$ is the coefficient for the linear interaction between risk factor *k* and age, $\gamma_{k}$ is the coefficient for the linear interaction between risk factor *k*=3,4 (HBG and smoking) and sex, and $\bar{x}_{kst}$ is the mean of risk factor *k* for sex *s* at age *t* in the Philippines. Note that we used HBG as a proxy for diabetes acknowledging that HBG on a single measurement is not recommended for clinical diagnosis of diabetes. We used the CVD baseline hazard rates for the Philippines ($\varphi_{s}\left( t \right)$) and the risk factor coefficients ($\beta_{k}$, $\delta_{k}$, $\gamma_{k}$) supplied by Globorisk.^1^ We estimated the age-sex-specific means of the risk factors ($\overline{x}_{kst}$) from the 2013 Philippines’ National Nutrition Survey (NNS) data that was nationally representative and more recent than the Globorisk sources. Values of the baseline hazards, coefficients, and means of risk factors are given below.

We used the age-year specific hazard rates obtained from (1) for age at the time of the survey (*t*=*j*) and at each of the 9 subsequent years of age to obtain the 10-year CVD risk for each participant:

$${Pr\left( CVD \right)}_{i}=1-\prod_{t=j}^{j+9} exp\left( -\varphi_{i}\left( t \right) \right) . (2)$$

Table S3.1. Baseline CVD event hazard rates by sex and age for the Philippines, $\varphi_{s}\left( t \right)$

| Age | 40-44 | 45-49 | 50-54 | 55-59 | 60-64 | 65-69 | 70-74 |
| --- | --- | --- | --- | --- | --- | --- | --- |
| Male | 0.00568 | 0.00940 | 0.01418 | 0.01981 | 0.02264 | 0.02361 | 0.02899 |
| Female | 0.00287 | 0.00488 | 0.00769 | 0.01109 | 0.01495 | 0.02005 | 0.02608 |

*Note*. Values shown for first year of the 10-year prediction period.

Table S3.2. Coefficients from Cox proportional hazard model of CVD event

| Risk factor main effect  ${(\beta}_{k})$ | | | | Risk factor interaction with age  ($\delta_{k}$) | | | | Risk factor interaction with sex  ($\gamma_{k}$) | |
| --- | --- | --- | --- | --- | --- | --- | --- | --- | --- |
| SBP | TC | HBG | SMK | SBP | TC | HBG | SMK | HBG | SMK |
| 0.3070 | 0.6149 | 1.4753 | 1.8467 | -0.0022 | -0.0069 | -0.0132 | -0.0221 | 0.4050 | 0.3254 |

Table S3.3. Age- and sex-specific means of risk factors ($\overline{x}_{kst}$)

|  | Systolic blood pressure (SBP), mmHG | | Total blood cholesterol (TC), mmol/L | | High blood glucose (HBG), % | | Current smoker  (SMK), % | |
| --- | --- | --- | --- | --- | --- | --- | --- | --- |
| Age | Female | Male | Female | Male | Female | Male | Female | Male |
| 40-44 | 116.7 | 121.1 | 5.21 | 5.24 | 3.9% | 6.4% | 5.4% | 48.4% |
| 45-49 | 121.1 | 124.0 | 5.47 | 5.27 | 6.8% | 7.7% | 7.1% | 47.0% |
| 50-54 | 125.7 | 126.5 | 5.80 | 5.23 | 10.6% | 7.8% | 9.2% | 43.7% |
| 55-59 | 127.4 | 128.5 | 6.06 | 5.18 | 9.6% | 12.0% | 8.5% | 48.7% |
| 60-64 | 130.3 | 131.8 | 5.99 | 5.27 | 12.4% | 15.0% | 10.0% | 44.4% |
| 65-69 | 132.8 | 133.9 | 5.96 | 5.06 | 11.8% | 10.1% | 13.0% | 34.8% |
| 70-74 | 132.6 | 134.9 | 5.80 | 5.16 | 9.1% | 6.5% | 14.8% | 30.1% |

**References**

1. Hajifathalian K, Ueda P, Lu Y, et al. R: A novel risk score to predict cardiovascular disease risk in national populations (Globorisk): a pooled analysis of prospective cohorts and health examination surveys. Lancet Diabetes Endocrinol. 2015 May;3(5):339–355.

**Supplementary Text S4. CVD risk concentration index**

We used the Erreygers concentration index (ECI)^1^ to measure absolute disparity in CVD risk (and in each risk factor used to predict this risk) over the distribution of the household wealth index. The wealth index is an ordinal and increasing measure of socioeconomic position. Consider a sample of *n* individuals ordered in increasing values of this index, $i=1,2,\ldots,n.$ Then, $r_{i}=\left( 2i-1 \right)/{2n}=\left( i-0.5 \right)/n$ is the fractional rank of individual $i$ in the distribution of the wealth index. Let $\mathbf{y}$ denote a vector containing either the CVD risks of the individuals $\left( y_{i} \right)$or the values of a risk factor. Then,

$ECI\left( y \right)=\frac{8}{y^{max}-y^{min}}Cov\left( y_{i},r_{i} \right)$

where $y^{max}$ is the maximum possible value of $y_{i}$ and $y^{min}$ is the minimum. When $y_{i}$ is bounded between 0 and 1, as it is for the predicted CVD risk $\left( {Pr\left( CVD \right)}_{i} \right)$ given by equations (1) and (2) in Supplementary Text S3, $ECI$ simplifies to $=8Cov\left( y_{i},r_{i} \right)$.

Using the relationship between covariance and bivariate linear least squares regression, we used the latter to estimate the *ECI*.^2^ We applied sample weights and adjusted the fractional ranks to take account of them.^2,3^ We also took account of ties, where two or more participants had the same wealth index value.^2^ Confidence intervals were adjusted for sample stratification and clustering. Minimum and maximum values of each risk factor are given in Table S4.1.

Table S4.1. Minimum and maximum values of CVD risk and each risk factor

| Risk factor | $y^{min}$ | $y^{max}$ |
| --- | --- | --- |
| CVD risk | 0 | 1 |
| High CVD risk | 0 | 1 |
| Age | 40 | 74 |
| SBP | 70 | 270 |
| TC | 1.75 | 20 |
| HBG | 0 | 1 |
| Smoking | 0 | 1 |
| Body Mass Index | 12 | 70 |
| Low physical activity | 0 | 1 |
| Fat share | 0 | 1 |
| Alcohol | 0 | 1 |

**References**

1. Erreygers GR. Correcting the Concentration Index. *J Health Econ*. 2009;28(2):504–515.
2. O’Donnell O, O’Neill S, Van Ourti T, Walsh B. Conindex: Estimation of concentration indices. *The Stata Journal* 2016;16:112-138.
3. Lerman RI, Yitzhaki S. Improving the accuracy of estimates of Gini coefficients. *J Econom* 1989;42:43-47.

**Supplementary Text S5. Decomposition of CVD risk concentration index**

The Erreygers concentration index (ECI)^1^ of CVD risk is the scaled covariance between that variable and the fractional rank in the wealth index distribution (Supplementary Text S4). Our objective was to identify the contribution of each risk factor to this measure of socioeconomic disparity in CVD risk. Because CVD risk is a nonlinear function of risk factors (Supplementary Text S3), the contribution of a risk factor to the ECI was not additively separable from the contributions of the other risk factors. Consequently, the order in which the contributions of the risk factors were calculated matters.

We used the Shapley value decomposition to deal with this problem.^2,3^ This method identified the contribution of a risk factor to the ECI by calculating the risk factor’s marginal contribution for each possible sequence of calculating the contributions of all risk factors and taking the average of marginal contribution over all possible sequences. The method has two main advantages. First, it is *path independent* in the sense that the contribution of a risk factor does not depend on the order in which the contributions of the other risk factors are calculated. Second, it is *exact*: the contributions of all risk factors sum to the ECI.

Let ${Pr\left( CVD \right)}_{i}$ be the CVD risk of individual *i*, which is a nonlinear function given by equations (1) and (2) in Supplementary Text S3 of four risk factors $x_{ki},k=1,\ldots,4$ (SBP, TC, HBG, and Smoking) and of sex and age. $ECI=8Cov\left[ {Pr(CVD)}_{i},r_{i} \right],$ where $r_{i}$ is the individual’s fractional rank in the wealth index distribution, as defined in Supplementary Text S4. We stratified by sex. For each sex, the ECI was a nonlinear function of the four risk factors, age, and $r_{i}$. Write this as $ECI=g\left( x_{1i},\ldots,x_{4i},{age}_{i},r_{i} \right)$.

For each sex, we used the *mean-equalized* Shapley value decomposition.^2,3^ This involved identification of the marginal contribution of a risk factor *k* through comparison of the ECI obtained using the actual value of that risk factor ($x_{ki}$) with the ECI obtained when that risk factor was set to its age-gender-specific mean value ($\bar{x}_{kst}$). Due to the nonlinearity of $g\left( x_{1i},\ldots,x_{4i}{,age}_{i},r_{i} \right),$ this marginal contribution depended on the values of the other risk factors and, thus, on the order in which the risk factors were mean-equalized. We therefore calculated the marginal contribution of a risk factor *k* for all possible sequences in which it and the other risk factors could be mean-equalized and took the average across all possible sequences.

With a set of 4 risk factors, there were 4!=24 different sequences in which they could be equalized. Let *m* be a particular permutation of these possible sequences. For example, *m*={3,1,2,4} corresponds to the case in which risk factor 3 (HBG) was equalized first, then risk factor 1 (SBP), then risk factor 2 (TC), and, finally, risk factor 4 (Smoking). For this permutation, the marginal contribution of risk factor 2, for example, was $\Delta{ECI}_{2}^{m}=$ $g\left( \bar{x}_{3},\bar{x}_{1},x_{2i},x_{4i},{age}_{i},r_{i} \right)-g\left( \bar{x}_{3},\bar{x}_{1},\bar{x}_{2},x_{4i},{age}_{i},r_{i} \right)$. The marginal contribution of risk factor 2 was then calculated by taking the average over these permutations, $\Delta{ECI}_{2}=\frac{1}{24}\sum_{m=1}^{24} \Delta{ECI}_{2}^{m}$.

Since the Globorisk equation used age- and sex-specific means of the risk factors (Supplementary Text S3), we equalized the risk factors by setting them to their age- and sex-specific means $\bar{x}_{kst}$ (not the overall mean $\bar{x}_{ks}$), and so calculated the contribution of each risk factor that was unrelated to age. The contribution of age was calculated as a residual to ensure that the contribution of all risk factors and age would add up to the *ECI*, i.e. $\Delta{ECI}_{age}= ECI-\sum_{k=1}^{4} \Delta{ECI}_{k}.$

**References**

1. Erreygers GR. Correcting the Concentration Index. *J Health Econ*. 2009;28(2):504–515.
2. Sastre M, Trannoy AR. Shapley inequality decomposition by factor components: Some methodological issues. *J Econ*. 2002;77(S1):51–89.
3. Davillas A, Jones AM. Ex ante inequality of opportunity in health, decomposition and distributional analysis of biomarkers. J Health Econ. 2020 Jan 1;69:102251.
4. Shorrocks, AF. Decomposition procedures for distributional analysis: a unified framework based on the Shapley value. *Journal of Economic Inequality 2013;11*(1): 99–126

**Supplementary Text S6. Adjustment to sample weights**

We applied sample weights in all analyses. We adjusted these weights to correct for differences between the full sample and the complete cases primary analysis sample in observable characteristics for which there was full item response for all participants $\left( Z \right)$. To do this, we defined a binary indicator of inclusion in the complete cases sample $\left( R_{i} \right)$and estimated a logistic regression model of this indicator on age-sex category indicators, educational attainment groups, and an urban/rural indicator, with the original sample weights $\left( w_{i} \right)$ applied.^1^ Let $Pr\left( R_{i}=1|Z_{i} \right)=\Lambda\left( Z_{i}\hat{\theta} \right)$ be the logit-estimated propensity score of inclusion in the complete cases sample. We adjusted the original sample weights by dividing them by this propensity score: $\tilde{w}_{i}=\frac{w_{i}}{\Lambda\left( Z_{i}\hat{\theta} \right)}.$ We rescaled the adjusted weights to have a mean of 1. We made an analogous adjustment to the sample weights for the complete cases secondary analysis sample.

**References**

1. Moore CG, Lipsitz SR, Addy CL, Hussey JR, Fitzmaurice G, Natarajan S. R: Logistic Regression With Incomplete Covariate Data in Complex Survey Sampling: Application of Reweighted Estimating Equations. Epidemiology. 2009 May;20(3):382–390.

**Supplementary Figure S1. Participant flow**


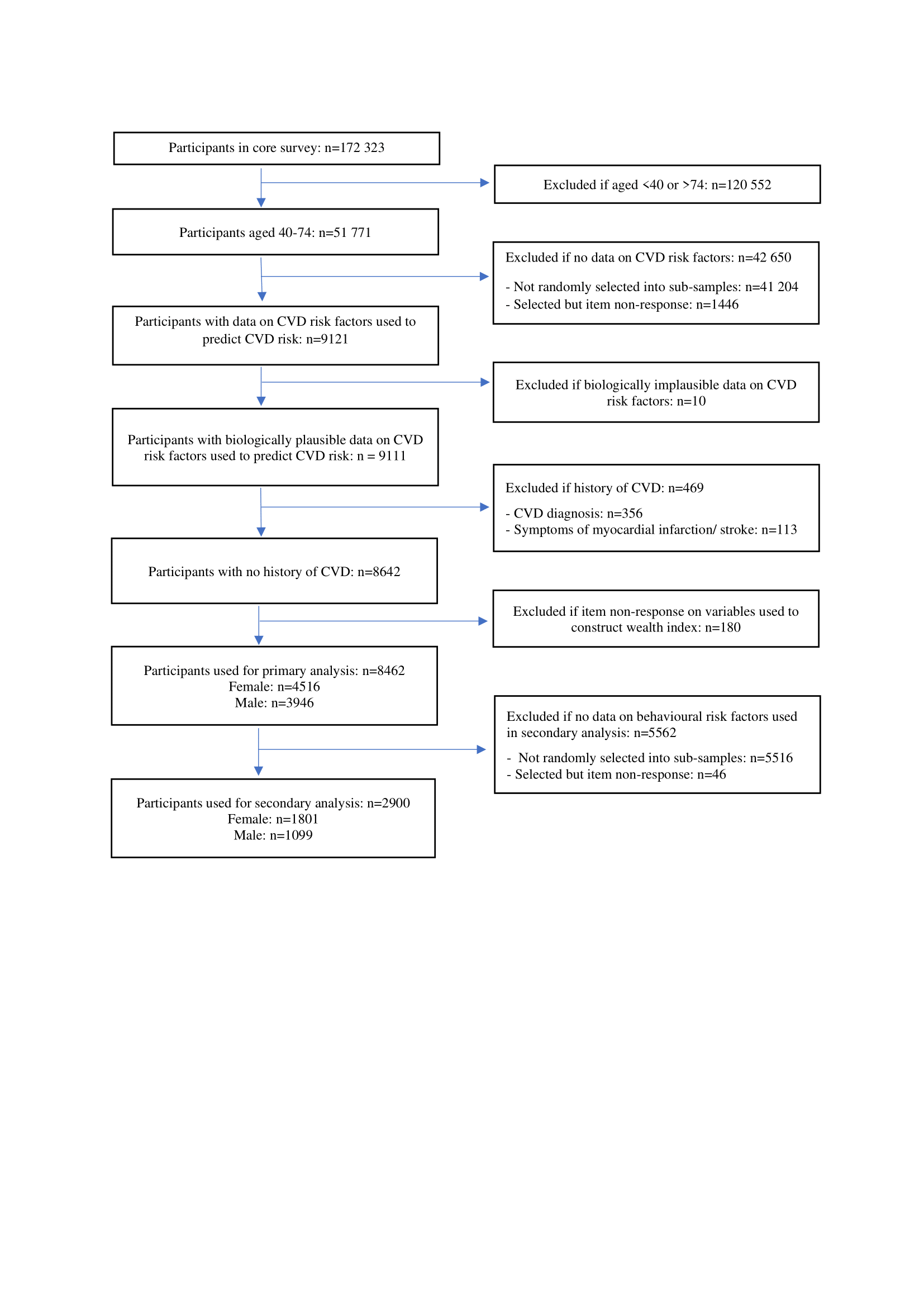


Participant flow. CVD risk factors used to predict CVD risk using Globorisk equation were age, sex, systolic blood pressure (SBP), total cholesterol (TC), fasting blood sugar (FBS), and smoking status. Participants may not have had data on SBP, TC, FBS, or smoking because they were not randomly selected into the respective sub-samples for whom these data were collected, or because they were selected but had item non-response. Biologically plausible values were: SBP [70-270] mmHg, TC [1.75-20.0] mmol/L, FBS [30-600] mg/dL. Symptoms of myocardial infarction / stroke if participant reported ever having experienced sudden weakness, heaviness, or numbness on one side or part of the body that lasted for more than 24 hours or sudden slurring of speech or inability to say what they wanted for more than 24 hours. Secondary analysis was the decomposition of inequality in SBP, TC, and high blood glucose into contributions of distal risk factors: body mass index (BMI), low physical activity, fat intake share of diet, and alcohol consumption. Plausible values of BMI were [12-70] kg/m2.

**Supplementary Figure S2. CVD risk by fatality, sex and wealth index quintile group**


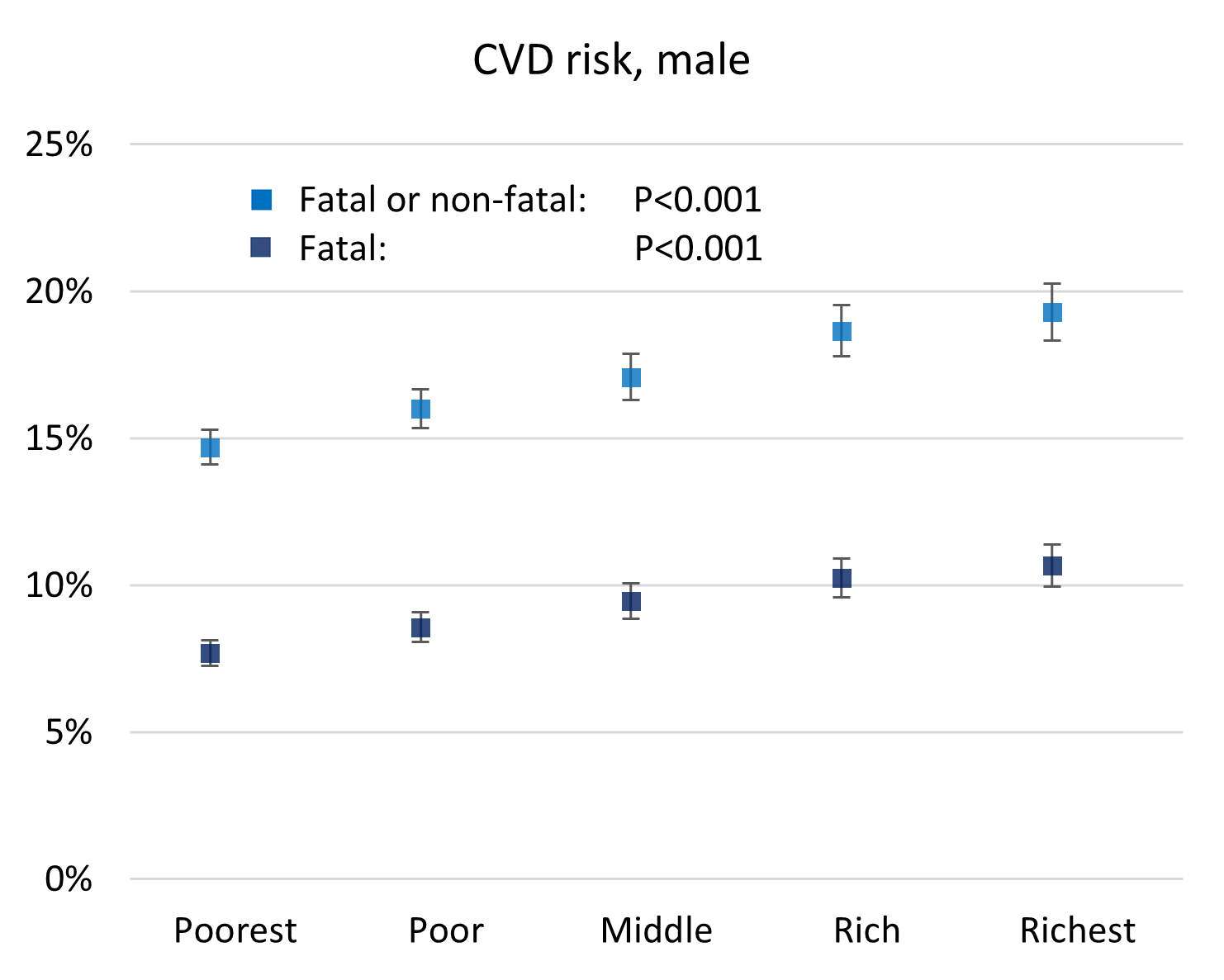

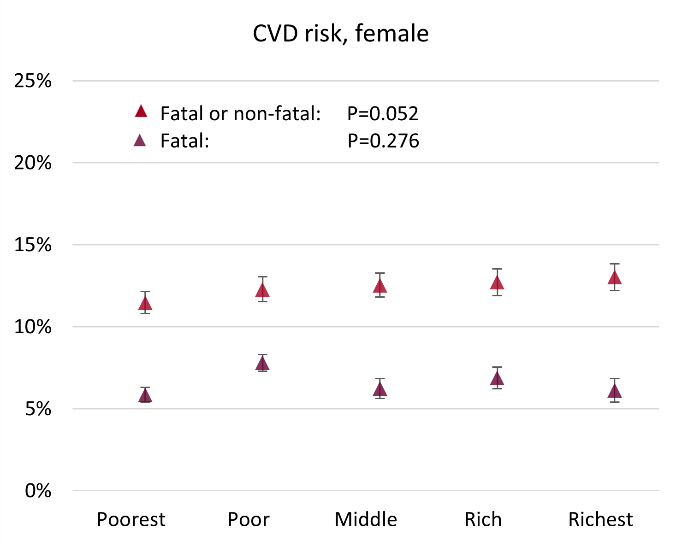


Mean predicted CVD risk by wealth index quintile group and sex. *Fatal and non-fatal* refers to the percent chance of a) death from ischaemic heart disease or stroke, b) a sudden cardiac death, or c) a non-fatal myocardial infarction or stroke within 10 years. *Fatal* refers to the percent chance of a) or b) within 10 years. Individuals aged 40-74 years. Females: n=4516. Males: n=3946. Whiskers show 95% confidence intervals. P values from tests of equal means across groups.

**Supplementary Figure S3. Decomposition of CVD risk by sex and urban/rural location**


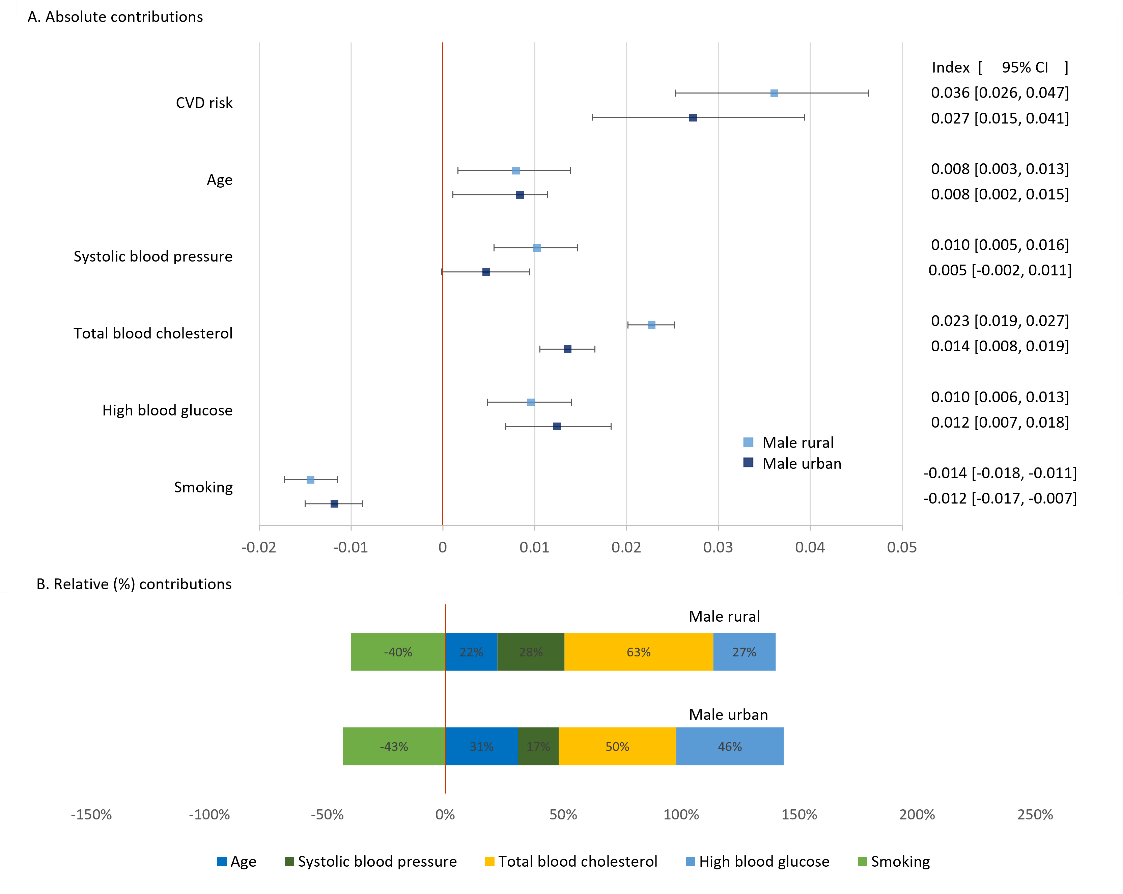

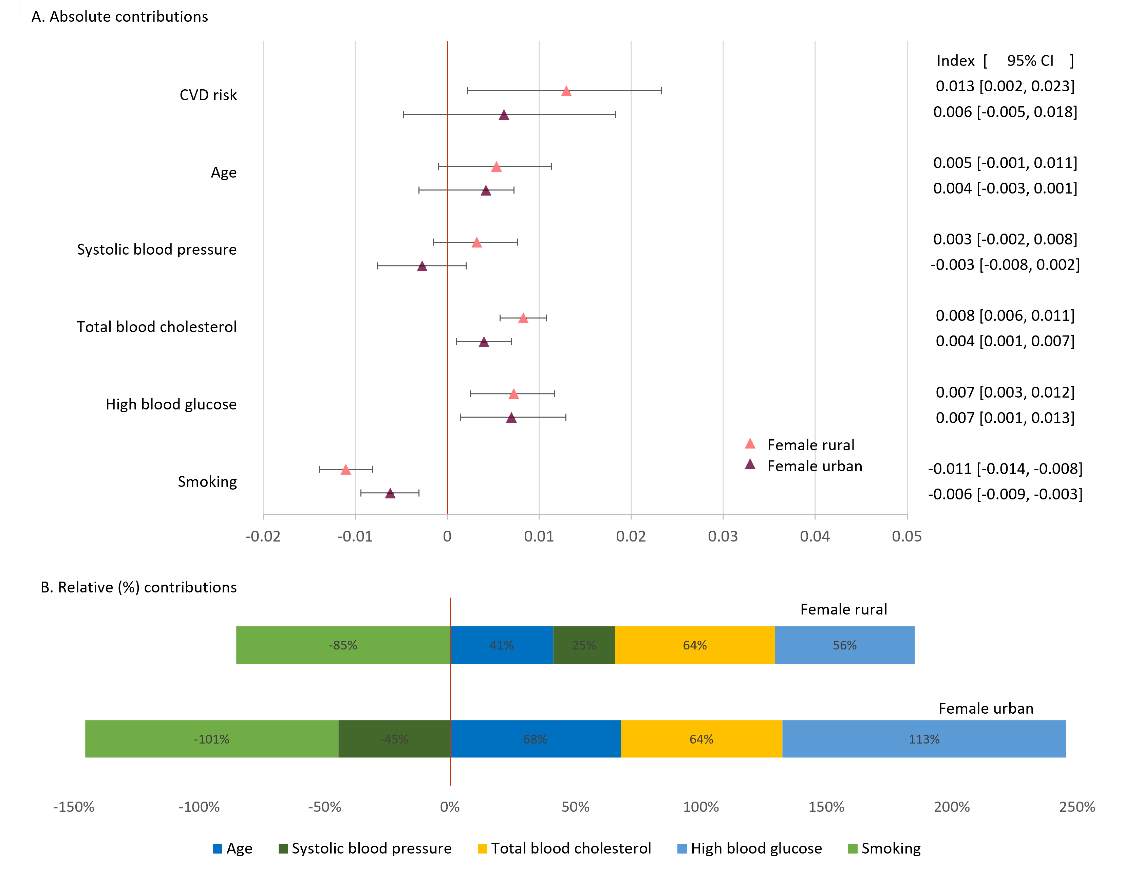


Females

Males

Decomposition of concentration index measure of socioeconomic disparity in CVD risk into contributions of risk factors by sex and urban/rural location. Top panel for each sex shows absolute contributions. Bottom panel for each sex shows these contributions as percentages of the concentration index. Female Urban: n=1957. Female Rural: n=2559. Male Urban: n=1571. Male Rural: n=2375. Whiskers show 95% confidence intervals obtain from a bootstrap with 1000 replications.

**Supplementary Figure S4. Decompositions of predicted SBP, TC and HBG by sex**


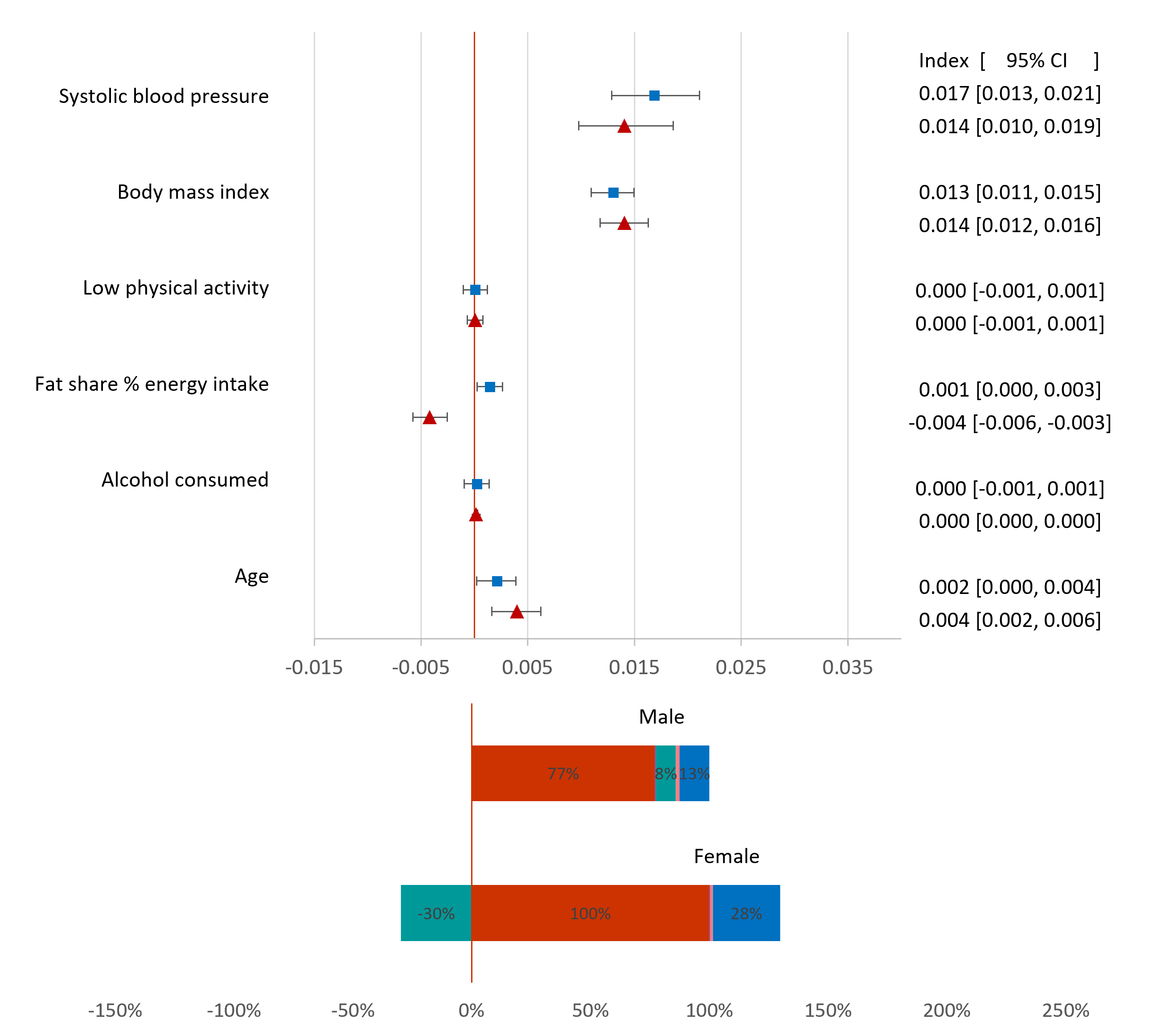

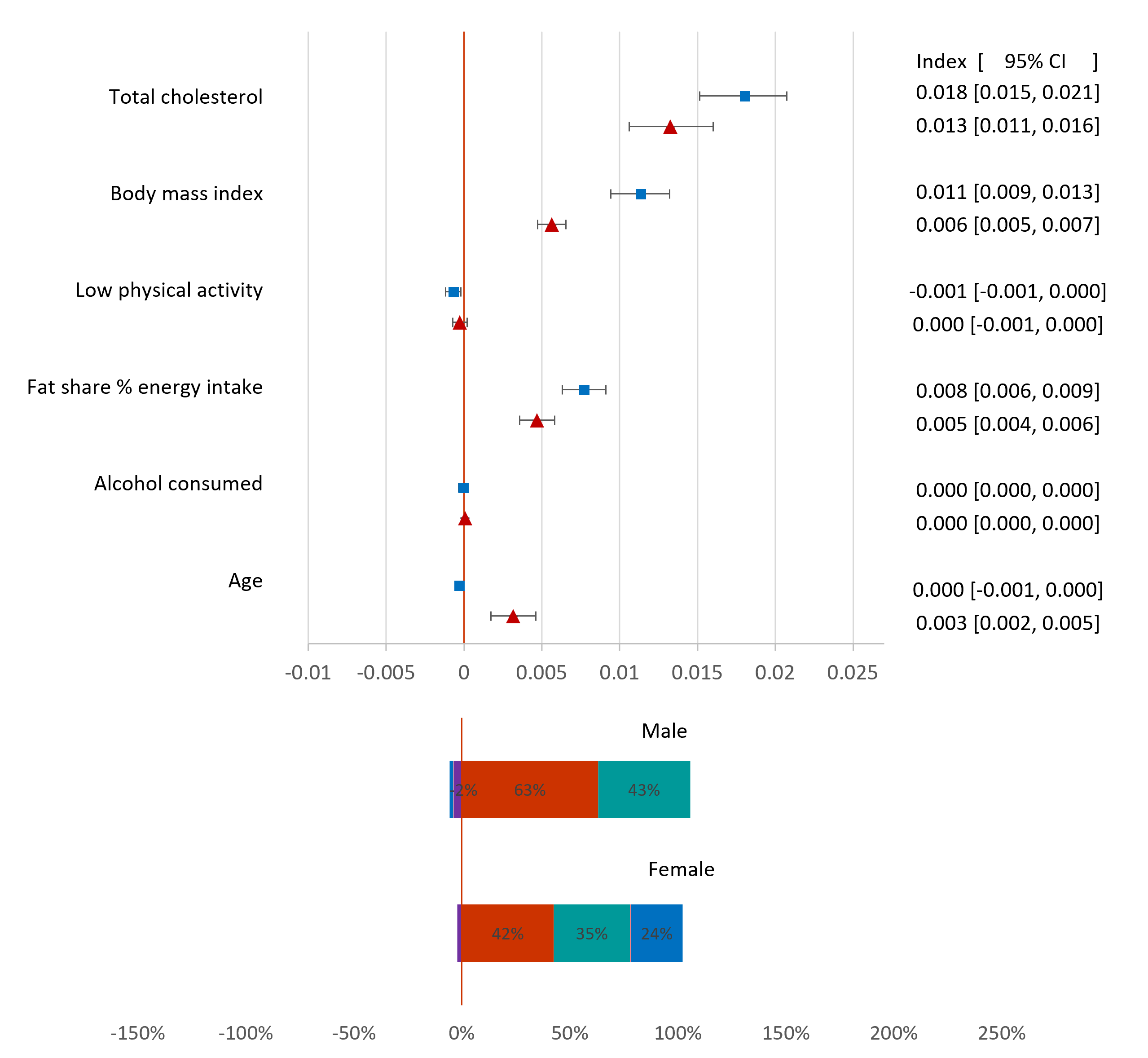

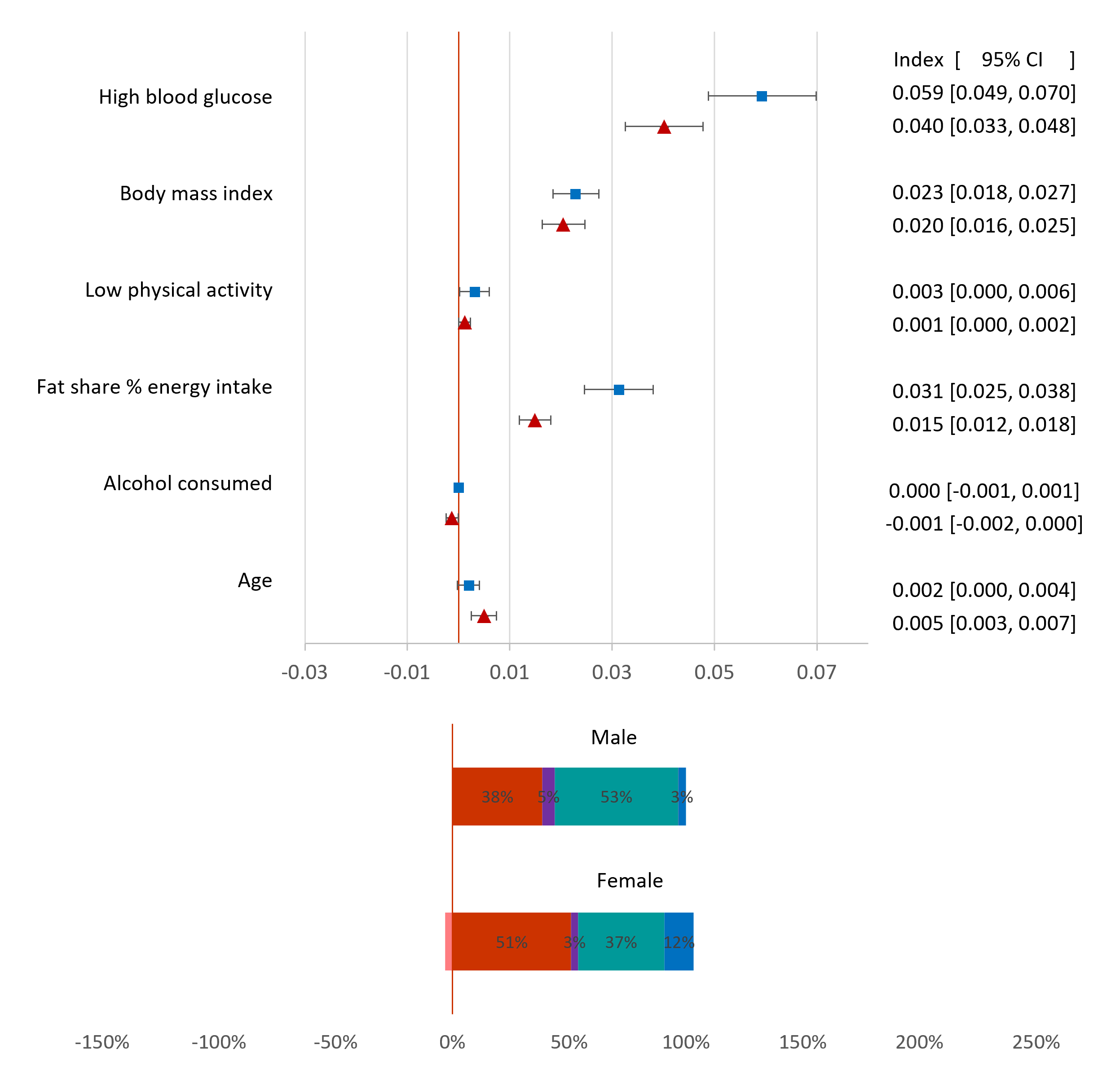

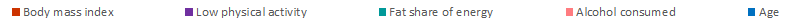

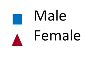

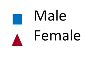

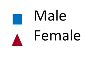


A. Predicted SBP

B. Predicted TC

C. Predicted HBG

Decomposition of concentration index measure of socioeconomic disparity in predicted SBP, TC, and HBG into contributions of distal risk factors. Females: n=1801. Males: n=1099. Whiskers show 95% confidence intervals obtain from a bootstrap with 1000 replications.

**Supplementary Table S1. Participant characteristics in NNS core survey and two analysis samples, participants aged 40-74 years**

|  |  | Core survey  sample | | Primary analysis sample | | Secondary analysis sample | |
| --- | --- | --- | --- | --- | --- | --- | --- |
|  |  | Female n=26,492 | Male n=25,279 | Female n=4516 | Male n=3946 | Female n=1801 | Male n=1099 |
| Age in years | Mean (SD) | 52.7 (9.2) | 52.1 (8.8) | 52.7 (9.2) | 52.1 (8.8) | 52.7 (9.2) | 52.1 (8.8) |
| Education, highest level | n  (%) |  |  |  |  |  |  |
| Primary education |  | 11,192 (38.9) | 10,906 (39.5) | 2,002 (38.9) | 1,872 (39.5) | 795 (38.9) | 483 (39.1) |
| Secondary education |  | 8,823 (34.9) | 8,412 (35.0) | 1,519 (34.9) | 1,289 (35.0) | 607 (35.0) | 359 (35.1) |
| Tertiary education |  | 6,477 (26.1) | 5,961 (25.5) | 995 (26.2) | 785 (25.5) | 399 (26.1) | 257 (25.7) |
| Location | n  (%) |  |  |  |  |  |  |
| Urban |  | 12,149 (52.4) | 11,204 (50.8) | 1,957 (52.4) | 1,571 (50.7) | 826 (52.2) | 509 (50.8) |
| Rural |  | 14,343 (47.6) | 14,075 (49.2) | 2,559 (47.6) | 2,375 (49.3) | 975 (47.8) | 590 (49.2) |

Note. The core survey sample includes all participants in the 2013 NNS. The table shows characteristics of participants aged 40-74 years. The primary analysis sample was used to measure and decompose disparity in CVD risk. The secondary analysis sample was used to measure and decompose disparity in SBP, TC, and HBG into contributions of BMI, low physical activity, fat intake, and alcohol consumption. See Supplementary Figure S1 for selection of the respective samples. Table shows characteristics with full item response in the core survey. Frequencies are unweighted. Means and percentages are after application of sample weights

**Supplementary Table S2. Average effect on predicted CVD risk of a unit-change in each proximal risk factor scaled by its range.**

| Male  n=3946 | Systolic blood pressure | Total  cholesterol | High  blood  glucose | Smoking |
| --- | --- | --- | --- | --- |
| Poorest | 20.4% | 15.7% | 6.2% | 3.3% |
| Poor | 22.5% | 17.0% | 6.7% | 3.7% |
| Middle | 24.3% | 18.2% | 6.9% | 4.0% |
| Rich | 26.4% | 19.9% | 7.4% | 4.4% |
| Richest | 27.4% | 20.6% | 7.4% | 4.8% |
| All | 24.2% | 18.3% | 6.9% | 4.0% |

| Female  n=4516 | Systolic blood pressure | Total  cholesterol | High  blood  glucose | Smoking |
| --- | --- | --- | --- | --- |
| Poorest | 14.9% | 12.3% | 7.8% | 4.8% |
| Poor | 16.0% | 13.1% | 8.0% | 5.5% |
| Middle | 16.4% | 13.5% | 8.5% | 5.6% |
| Rich | 16.7% | 13.8% | 8.3% | 5.8% |
| Richest | 17.0% | 14.2% | 8.2% | 6.1% |
| All | 16.2% | 13.4% | 8.1% | 5.5% |

*Note*. Estimates of the average effect of a unit-change in each proximal risk factor on predicted CVD risk scaled by its range. The estimated effects were averaged over the sample and within each wealth quintile group. The effect on predicted CVD risk is given in percentage points. Males: n=3946. Females: n=4516.

**Supplementary Table S3. Mean CVD risk, risk factors and socioeconomic status by sex and urban/rural location**

|  |  | | Female | |  |  | | Male | |  |
| --- | --- | --- | --- | --- | --- | --- | --- | --- | --- | --- |
|  | Urban  n=1957 | | Rural  n=2559 | | Urban=Rural  n=4516 | Urban  n=1571 | | Rural  n=2375 | | Urban=Rural  n=3946 |
|  | Mean | [95% CI] | Mean | [95% CI] | P-value | Mean | [95% CI] | Mean | [95% CI] | P-value |
| CVD risk, % | 12.7 | [12.2, 13.2] | 12.1 | [11.7, 12.6] | 0.0974 | 18.2 | [17.6, 18.8] | 16.1 | [15.7, 16.5] | <0.001 |
| High CVD risk >20% | 18.8 | [17.0, 20.5] | 17.8 | [16.3, 19.2] | 0.3875 | 35.9 | [33.5, 38.2] | 27.5 | [25.7, 29.3] | <0.001 |
| Age, years | 52.9 | [52.5, 53.3] | 52.6 | [52.2, 52.9] | 0.239 | 52.0 | [51.6, 52.5] | 52.2 | [51.8, 52.5] | 0.6172 |
| Systolic blood pressure, mmHg | 124.4 | [123.5, 125.3] | 124.3 | [123.5, 125.2] | 0.9257 | 127.7 | [126.7, 128.7] | 125.1 | [124.3, 125.9] | <0.001 |
| Total blood cholesterol, mmol/L | 5.8 | [5.7, 5.8] | 5.5 | [5.5, 5.6] | <0.001 | 5.4 | [5.4, 5.5] | 5.0 | [5.0, 5.1] | <0.001 |
| High blood glucose,  FBS > 126 mg/dl | 9.4 | [8.1, 10.7] | 7.2 | [6.2, 8.2] | 0.0082 | 11.5 | [9.9, 13.1] | 6.1 | [5.2, 7.1] | <0.001 |
| Current smoker, % | 7.6 | [6.4, 8.8] | 9.5 | [8.4, 10.7] | 0.0184 | 42.7 | [40.3, 45.2] | 47.5 | [45.5, 49.5] | 0.0026 |
| Wealth index quintile group, % |  |  |  |  |  |  |  |  |  |  |
| Poorest | 9.2 | [8.0, 10.5] | 31.8 | [30.0, 33.6] | <0.001 | 9.1 | [7.7, 10.5] | 31.3 | [29.4, 33.1] | <0.001 |
| Poor | 14.4 | [12.8, 15.9] | 26.2 | [24.5, 27.9] | <0.001 | 14.4 | [12.7, 16.2] | 25.7 | [24.0, 27.5] | <0.001 |
| Middle | 20.3 | [18.5, 22.1] | 19.7 | [18.2, 21.2] | 0.5944 | 19.2 | [17.3, 21.2] | 20.8 | [19.2, 22.4] | 0.212 |
| Rich | 25.6 | [23.6, 27.5] | 13.9 | [12.6, 15.2] | <0.001 | 26.3 | [24.1, 28.5] | 13.5 | [12.2, 14.9] | <0.001 |
| Richest | 30.5 | [28.4, 32.5] | 8.4 | [7.3, 9.4] | <0.001 | 31.0 | [28.7, 33.3] | 8.7 | [7.5, 9.8] | <0.001 |

**Supplementary TableS4. Averaged marginal effects (AME) of distal risk factors and age on systolic blood pressure, total cholesterol, and probability of high blood glucose by sex**

|  | Systolic blood pressure (mm/Hg) | | Total cholesterol (mmol/L) | | High blood glucose  (FBS>126 mg/dl) | |
| --- | --- | --- | --- | --- | --- | --- |
| Female | n=1801 | | n=1801 | | n=1801 | |
|  | AME | [95% CI] | AME | [95% CI] | AME | [95% CI] |
| Body mass index | 0.522 | [0.397, 0.648] | 0.202 | [0.130, 0.274] | 0.036 | [0.023, 0.050] |
| Low physical activity | 0.022 | [-0.180, 0.224] | -0.019 | [-0.128, 0.090] | -0.003 | [-0.030, 0.024] |
| Fat share in energy | -1.718 | [-2.996, -0.440] | 0.543 | [-0.167, 1.253] | 0.154 | [0.057, 0.252] |
| Any alcohol last month | -0.041 | [-0.347, 0.266] | -0.009 | [-0.164, 0.146] | 0.048 | [0.013, 0.083] |
| Age 45-49 | 0.554 | [0.250, 0.858] | 0.321 | [0.174, 0.469] | 0.043 | [-0.003, 0.089] |
| Age 50-54 | 1.126 | [0.747, 1.505] | 0.680 | [0.534, 0.826] | 0.065 | [0.031, 0.100] |
| Age 55-59 | 1.062 | [0.733, 1.390] | 0.977 | [0.702, 1.252] | 0.062 | [0.032, 0.093] |
| Age 60-64 | 1.583 | [1.191, 1.974] | 0.899 | [0.704, 1.094] | 0.100 | [0.049, 0.151] |
| Age 65-69 | 1.816 | [1.413, 2.220] | 1.051 | [0.799, 1.302] | 0.091 | [0.032, 0.151] |
| Age 70-74 | 1.890 | [1.508, 2.272] | 0.791 | [0.603, 0.979] | 0.063 | [0.016, 0.111] |
|  |  | |  | |  | |
| Male | n=1099 | | n=1099 | | n=1099 | |
|  | AME | [95% CI] | AME | [95% CI] | AME | [95% CI] |
| Body mass index | 0.434 | [0.274, 0.595] | 0.371 | [0.285, 0.458] | 0.038 | [0.017, 0.058] |
| Low physical activity | 0.136 | [-0.123, 0.394] | 0.001 | [-0.160, 0.162] | 0.019 | [-0.024, 0.062] |
| Fat share | 0.500 | [-0.962, 1.962] | 1.420 | [0.688, 2.152] | 0.349 | [0.152, 0.547] |
| Any alcohol last month | 0.450 | [0.188, 0.712] | -0.087 | [-0.233, 0.059] | 0.004 | [-0.032, 0.039] |
| Age 45-49 | 0.304 | [-0.057, 0.665] | 0.002 | [-0.232, 0.237] | -0.017 | [-0.074, 0.039] |
| Age 50-54 | 0.484 | [0.077, 0.891] | 0.090 | [-0.136, 0.315] | 0.016 | [-0.040, 0.071] |
| Age 55-59 | 0.909 | [0.500, 1.318] | -0.016 | [-0.260, 0.228] | 0.061 | [0.013, 0.108] |
| Age 60-64 | 1.094 | [0.672, 1.516] | 0.293 | [0.000, 0.586] | 0.120 | [0.051, 0.189] |
| Age 65-69 | 1.541 | [1.106, 1.976] | -0.187 | [-0.478, 0.104] | 0.050 | [-0.028, 0.128] |
| Age 70-74 | 1.528 | [0.995, 2.060] | 0.230 | [-0.144, 0.604] | 0.003 | [-0.070, 0.077] |

*Note*. Estimates of the effect of a standard deviation increase in each distal risk factor (BMI, low physical activity, far share in energy intake and any alcohol consumption in the last month) on systolic blood pressure (SBP), total cholesterol TC), and the probability of high blood glucose (i.e. FBS > 126 mg/dl). For age, effect of a change from the reference group aged 40-44 years. The estimated marginal effects (ME) were averaged over the respective sample. Estimates for SBP and TC obtained from generalised linear models, each with a Poisson distribution and log link function. Estimates for diabetes obtained from logit model. 95% confidence intervals in brackets.
